# Comparative Evaluation of Nanosized Intracanal Medicaments on the Penetration and Fracture Resistance of Root Dentin: An In Vitro Study

**DOI:** 10.7759/cureus.89192

**Published:** 2025-08-01

**Authors:** Mayakiru Pakyntein, Suchismita Chakraborty, Rubi Kataki, Adrija Deka, Debosmita Roy, Diganta Borah

**Affiliations:** 1 Conservative Dentistry and Endodontics, Regional Dental College, Guwahati, IND

**Keywords:** calcium hydroxide, chitosan, nanoparticles, scanning electron microscope, silver

## Abstract

Introduction: The use of calcium hydroxide (CH) is crucial in the proper disinfection of root canals. However, CH affects the fracture resistance of root dentine and also has limited permeability. Nano-based intracanal medicaments have several benefits over traditional medicaments, including a greater surface area-to-volume ratio and better penetrability. Therefore, this in vitrostudy was performed to evaluate and compare the effect of conventional CH and various nanoparticle-based intracanal medicaments on the penetration and fracture resistance of radicular dentin.

Materials and methods: One hundred samples were taken and randomly divided into four (n=20) experimental groups (nano-CH (NCH), nano-chitosan (NCS), NCH + nano-silver (NAg), NCS + NAg, and a control group (CH). At the end of one month, out of the 20 samples from each group, 10 samples were used to assess the fracture resistance in a universal testing machine, and the other 10 samples were used to determine the depth of penetration using a field emission scanning electron microscope.

Results: The mean fracture resistance of root dentin value was the highest with group II (NCS) and the least in the control group (CH), which was statistically significant. The maximum penetration depth of intracanal medicaments was observed with group III (NCH + NAg) and the least in the control group (CH), which was statistically significant. Among the thirds of the root samples, the highest mean depth of penetration was seen in the coronal third and the least in the apical third in all the groups, which was statistically significant.

Conclusion: The resistance to fracture and penetration depth at a four‑week interval were higher in nano forms when compared with conventional CH.

## Introduction

Eliminating all germs from the root canal system is the main goal of root canal therapy to stop reinfection and achieve full periradicular healing [1]. The intricate structures of the root canal make it difficult for adequate instrumentation, irrigation, and medications to get rid of the microbes that infiltrate the dentinal tubules to depths ranging from 200 to 1500 μm. This makes it more difficult for the host's defense cells and systemically administered antibiotics to work [2]. Intracanal medication is required due to the polymicrobial nature of endodontic infections [3,4].

Calcium hydroxide (CH), which is the most commonly used intracanal medicament, is highly alkaline and has a size range from 1 to 10 mm. It has properties including antimicrobial activity by changing the lipopolysaccharides of bacteria present in the cell wall and the ability to induce complex tissue formation primarily through its high pH and the release of calcium and hydroxyl ions, which promote mineralization [5]. Hence, for an intracanal medicament to have a positive impact, the medicament must come in contact with the pathogens in the dentinal tubules [6].

However, conventional medicaments have restricted permeability into the tubules (diameter ranges from 1 to 10 mm), which reduces their antibacterial property. Additionally, earlier research has demonstrated that the application of CH medicament for a long time has a detrimental effect on the resistance of teeth to fracture and reduces the microhardness of root dentin in cases of chronic infection, resorptive defects, and immature apices [7]. Several medications, including nanoparticles, have been proposed or used as a substitute for CH [8].

Nanoparticles are nanosized particles with a size of <100 nm, having unique properties such as ultra-small size, a larger surface area-to-volume ratio, high chemical reactivity, and greater interaction with the bacterial cells. The intracanal medicaments, in their nano forms, can penetrate the dentinal tubules (diameter of the dentinal tubules near the pulp is 2 mm) and reach the intricate areas of the root canal system, not only rendering the microorganisms inactive in the dentinal tubules but also acting as a barrier, preventing microbial recolonization [1,3].

Nano-CH (NCH) possesses several advantages over conventional CH, such as better penetration into dentinal tubules and a greater concentration than CH [1]. The antimicrobial activity of NCH, such as its effectiveness against *Enterococcus faecalis*, was superior to conventional CH [1].

Nano-chitosan (NCS) has a greater surface area and is positively surface charged, which interacts with the negatively charged bacterial cell surface, causing cell death. NCS tends to break down the structure of biofilms and decrease the number of bacteria that produce them. The increased surface area of NCS, which results in improved contact with the bacterial cell membrane, may be the cause of its enhanced antibacterial effectiveness [9].

Nano-silver (NAg) is frequently utilized because of its high bactericidal capability against multidrug-resistant bacteria as well as Gram-negative and Gram-positive bacteria. Silver can interact with the bacterial cell wall, changing its structure and causing tissue protein damage [10]. Of these nanoparticles, NAg has attracted the most attention because of its many medical applications. NAg has been shown to have an antibacterial effect on bacterial species such as *Staphylococcus aureus*, *Escherichia coli*, *Pseudomonas aeruginosa*, *Staphylococcus epidermidis*, and *Enterococcus faecalis* [11].

Various studies have demonstrated the penetration depth and resistance of root dentin to fracture for traditional intracanal medicament [3,7]. Nevertheless, there is no documented research on the combination of NCH and NCS with NAg (NCH + NAg, NCS + NAg). Hence, this in vitro study was carried out to assess the effect of CH, NCH, NCS, NCS + NAg, and NCH + NAg as intracanal medications on the penetration depth and resistance to fracture of root dentin after four weeks.

## Materials and methods

The ethical clearance for the study was obtained from the Institutional Ethics Committee of Regional Dental College, Guwahati, India (approval number: RDC/29/2011/Pt.I-/1738).

Sample preparation

One hundred extracted single‑rooted, single-canal mandibular premolar teeth previously screened by preoperative radiographs and fully formed apices were included in the study. No human participants were involved in this study. To maintain a standard root length of 14 mm in all the groups, the teeth were decoronated at the cementoenamel junction using a diamond disc. To determine the working length, a size 10 K-file (Dentsply Maillefer, Ballaigues, Switzerland) was used. The tip of the file was visualized at the apical foramen, and 0.5 mm was subtracted from this length. The root canals were mechanically prepared using Ni-Ti rotary files (Neoendo Orikam, India) up to a size of 30 (0.04° taper). Along with instrumentation, the canals were irrigated with 3% sodium hypochlorite (2 ml) between each succeeding file. Furthermore, 17% EDTA was used as a final irrigant after instrumentation (Dent Wash‑Prime Dental, India) for 3 min and then with saline (5 ml) after instrumentation. Sterile paper points were used to dry the canals. After preparation of the root canal, resin was used to seal the apical tip of the canal.

Group Distribution

The samples (n=100) were divided randomly into four experimental groups and a control group according to intracanal medicaments used: control (n=20), CH; Group I (n=20), NCH; Group II (n=20), NCS; Group III (n=20), NCH + NAg; and Group IV (n=20), NCS + NAg.

Medicament Application

For all groups, including the control group and nano-sized intracanal medicaments (Nano Research Elements, Haryana), a powder will be mixed with saline at a 1:1 powder-to-liquid ratio using a stainless steel spatula on a glass slab to achieve a paste consistency and then placed into the root canal. The canal spaces were filled with the medicament with the help of a lentulo spiral in a slow-speed handpiece (Mani Inc., Japan) and packed in the root canal to the level of the canal orifice.

The canal orifices of all teeth were sealed with a 3 mm thickness of Cavit. The specimens were then kept in an incubator at 37°C at 100% humidity (to simulate the moist environment of the oral cavity and prevent dehydration of the tooth samples) for four weeks. From the 20 samples from the individual group, 10 samples were randomly selected to check the fracture resistance in a universal testing machine (UTM), and 10 samples were used to check the penetration depth using a field emission scanning electron microscope (FESEM) at the end of the incubation period.

Sample evaluation for fracture resistance

**Figure 1:**
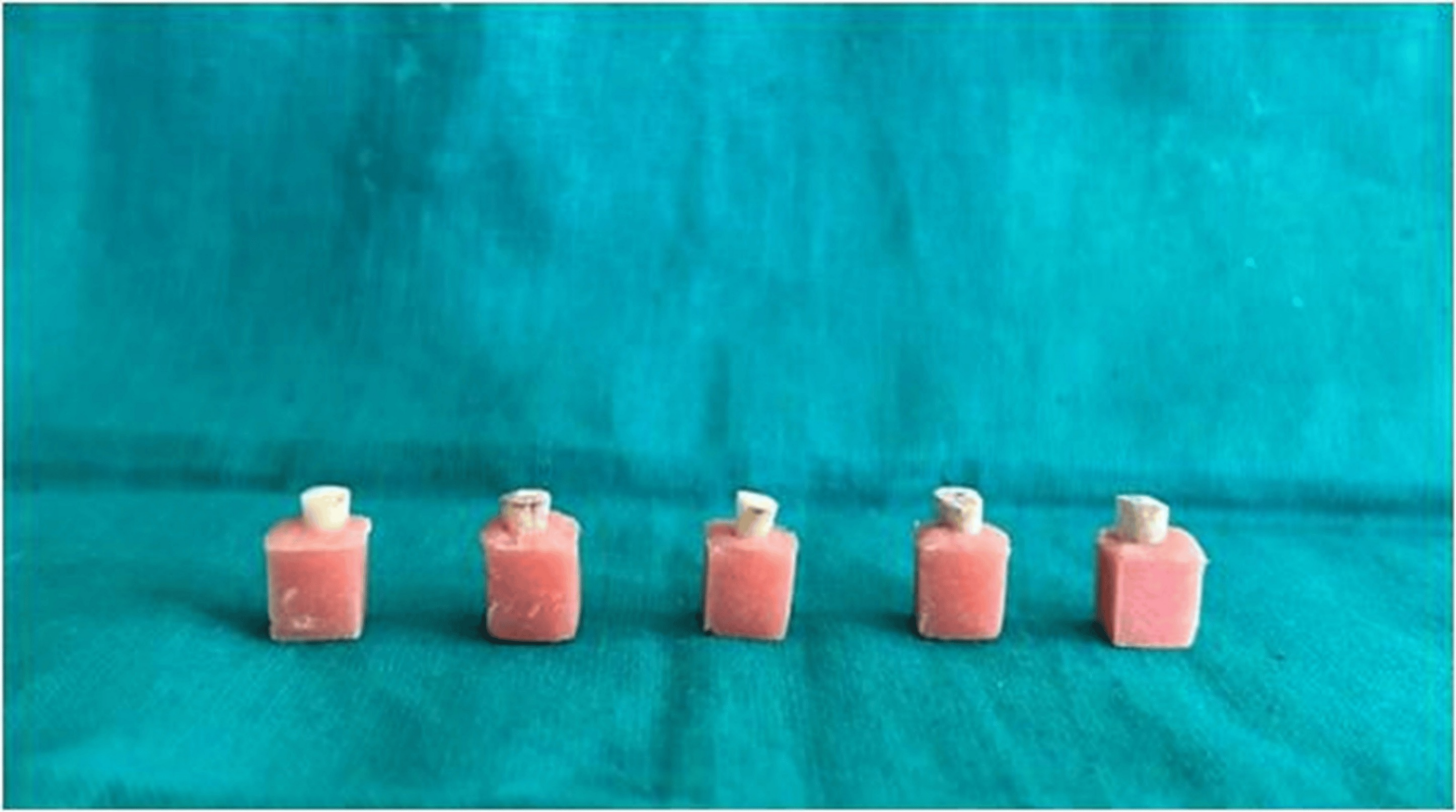
Representative image showing samples embedded in self-cure acrylic

The temporary restoration was removed from all the samples. Samples were placed directly in self-cure acrylic, exposing 3 mm of the coronal third of the root structure unhindered (Figure 1). A UTM was used to measure the fracture resistance of the teeth. Each specimen was subjected to a vertical load that exerted a compressive force at a crosshead speed of 0.5 mm/min. Newtons were used to test the teeth's resistance to breakage.

Sample evaluation for depth of penetration

**Figure 2:**
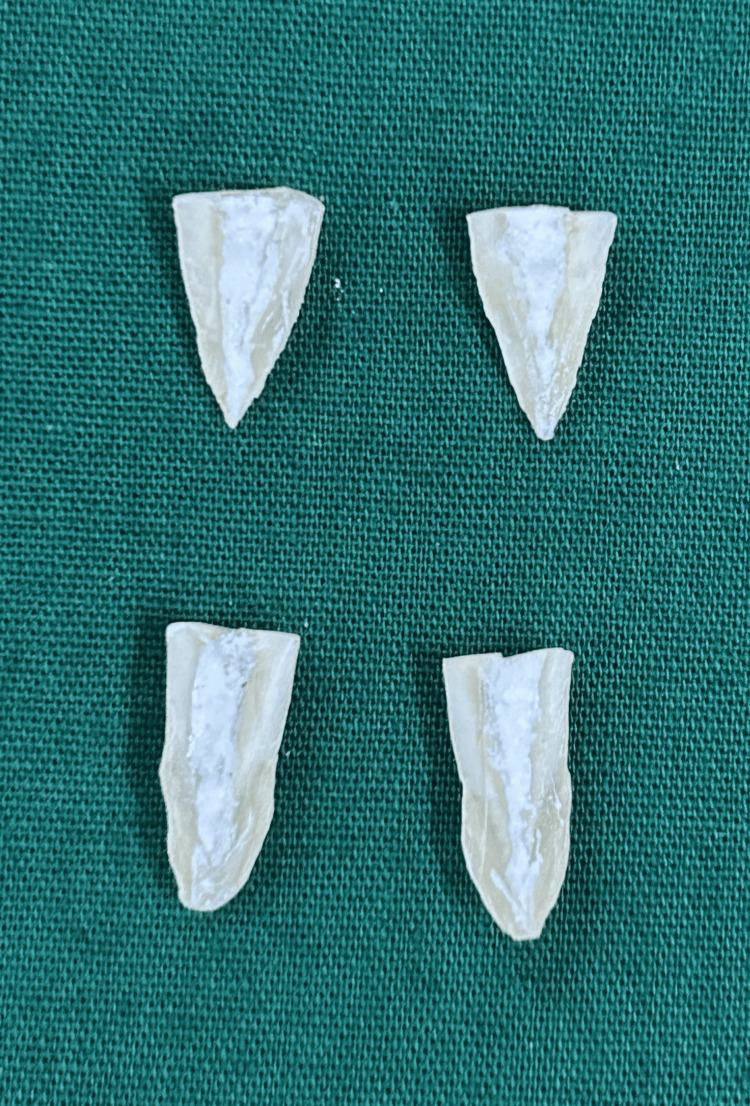
Representative image of the samples showing splitted roots using chisel

Using a diamond disc, longitudinal grooves were made on the roots, and they were divided into halves with the help of a chisel (Figure 2). Following dehydration, the specimens were mounted on a stub with a label and sputter-coated with gold. The FESEM (GeminiSEM 300, Zeiss) was used to scan every segment. A calibrated measuring instrument built into the microscope was used to assess the penetration depth of the intracanal medications in the coronal, middle, and apical thirds of the root canal at a 600X magnification.

Statistical Analysis

The data for the mean values and standard deviation for depth of penetration and the fracture resistance of teeth were determined, and the results were statistically analyzed using Tukey's post hoc test and one-way analysis of variance (ANOVA). One-way ANOVA was used to evaluate intergroup differences. Tukey's post hoc test was used to evaluate intragroup differences. The significance level was set at p < 0.05.

## Results

Fracture resistance of root dentin

Table 1 shows that the value for mean fracture resistance of root dentin was the highest with Group II (NCS) - 4.76 N, followed by Group IV (NCS + NAg) - 0.49 N, Group I (NCH) - 0.14 N, and Group III (NCH + NAg) - 0.06N. Control group (CH) - 0.05 N demonstrated the minimum fracture resistance, which was highly significant.

**Table 1:** Mean and SD value of fracture resistance of root dentin among experimental groups * Statistically significant at p < 0.05 SD: standard deviation, CH: calcium hydroxide, NCH: nano-calcium hydroxide, NCS: nano-chitosan, NAg: nano-silver

Compressive strength (kN /mm)	Mean ± SD	Group comparisons (p-values)			
		Control	Group I	Group II	Group III
Control (CH)	0.05 ± 0.01				
Group I (NCH)	0.14 ± 0.23	0.237			
Group II (NCS)	4.76 ± 1.98	<0.001*	<0.001*		
Group III (NCH + NAg)	0.06 ± 0.02	0.605	0.258	<0.001*	
Group IV (NCS + NAg)	0.49 ± 0.39	0.006*	0.062	<0.001*	0.007*

Depth of penetration of intracanal medicament

Group III (NCH + NAg) - 642.3 µm shows the greatest depth of penetration of intracanal medicaments, followed by Group I (NCH) - 586.1 µm, Group II (NCS) - 552.0 µm, and Group IV (NCS + NAg) - 546.8 µm. The least is seen in the control group (CH) - 355.2 µm, which was statistically significant (Table 2). Among the thirds of the root samples, the lowest mean depth of penetration was seen in the apical third, followed by the middle third, and the most significant penetration depth was observed in the coronal third in all the groups, which was statistically significant (Table 3).

**Table 2:** Comparison of the mean value of depth of penetration among experimental groups * Statistically significant at p < 0.05 SD: standard deviation, CH: calcium hydroxide, NCH: nano-calcium hydroxide, NCS: nano-chitosan, NAg: nano-silver

Depth of penetration	Mean ± SD	Group comparisons (p-values)			
		Control	Group I	Group II	Group III
Control (CH)	355.2 ± 86.3				
Group I (NCH)	586.1 ± 170.8	<0.001*			
Group II (NCS)	552.0 ± 146.8	<0.001*	0.002*		
Group III (NCH + NAg)	642.3 ± 146.2	<0.001*	<0.001*	<0.001*	
Group IV (NCS + NAg)	546.8 ± 138.1	<0.001*	<0.001*	0.392	<0.001*

**Table 3:** Comparison of the mean value of depth of penetration in coronal, middle, and apical section among different groups * Statistically significant at p < 0.05 SD: standard deviation, CH: calcium hydroxide, NCH: nano-calcium hydroxide, NCS: nano-chitosan, NAg: nano-silver

Root	Groups	Mean depth of penetration ± SD	Group comparisons (p-values)			
			Control	Group I	Group II	Group III
Coronal	Control (CH)	452.0 ± 28.9				
	Group I (NCH)	750.9 ± 25.5	<0.001*			
	Group II (NCS)	665.0 ± 32.3	<0.001*	<0.001*		
	Group III (NCH + NAg)	762.0± 25.8	<0.001*	0.339	<0.001*	
	Group IV (NCS + NAg)	662.2 ± 25.2	<0.001*	<0.001*	0.65	<0.001*
Middle	Control (CH)	358.2 ± 29.7	<0.001*	0.434	<0.001*	<0.001*
	Group I (NCH)	648.4 ± 22.9				
	Group II (NCS)	638.4 ± 29.1	<0.001*			
	Group III (NCH + NAg)	722.1 ± 14.4	<0.001*	<0.001*		
	Group IV (NCS + NAg)	619.8 ± 12.4	<0.001*	0.007*	0.079	
Apical	Control (CH)	255.5 ± 28.5	<0.001*	0.712	<0.001*	<0.001*
	Group I (NCH)	359.2 ± 32.7				
	Group II (NCS)	352.5 ± 30.2	<0.001*			
	Group III (NCH + NAg)	442.8 ± 28.5	<0.001*	0.001*		
	Group IV (NCS + NAg)	358.5 ± 23.1	<0.001*	0.959	0.669	

## Discussion

The prime objective of endodontic therapy is to treat infections of the dental pulp and periradicular area and promote periapical healing. Intracanal medications play a vital role in root canal disinfection since they stay in the canal for prolonged periods of time, depending on the clinical requirements, usually one to four weeks. They exert antimicrobial action; therefore, they effectively inhibit root canal infections. However, prolonged exposure to these medicaments has been observed to adversely affect the root dentin [12].

In dentistry, nanotechnology has recently aided in the creation of superior biomaterials with distinctive chemical, biological, and physical characteristics. Because of their greater surface-to-volume ratio and smaller dimension of the particle (<100 nm), which produces direct contact with the microorganisms and a highly reactive surface, the nanoparticles exhibit superior antibacterial properties. They then enter the dentinal tubules and, at lower dosages, have a long-lasting antibacterial action at the infection site [13].

In the present study, NCS demonstrated enhanced resistance to fracture and toughness, with particles displaying greater affinity for the collagenase enzyme, hence augmenting long-term dentin stability, which may have resulted in superior resistance to fracture in the NCS group in comparison to other groups, which is in accordance with previous research [3].

This current study found that the greatest depth of intracanal medication penetration was seen in Group III (NCH + NAg) with a mean penetration depth of 642.3 μm. This can be described by the fact that dentinal tubules, which have a width of roughly 2400-4280 nm, are substantially larger than nanoparticles below 200 nm. Following the findings of Sireesha et al., the current study's results demonstrated that NCH had a deeper penetration than CH [9].

The total eradication of microorganisms from the root canal is challenging due to the intricacies of root canal anatomy and inaccessibility to the devices and irrigating solutions employed. With reduced permeability into the dentinal tubules, the antibacterial activity of conventional medications is diminished [9]. According to studies, particles with a smaller size exhibit greater antibacterial activity than those with a larger size [1].

According to a study on the size and shape of CH particles, the researchers concluded that the particles become rounder as their length decreases and more rectangular as their length increases. Therefore, shorter particles are likely preferable for deep dentin penetration [1]. Thus, reducing the size of the CH particles and producing them in nanoform may improve the intracanal medication's penetrability into the dentinal tubules and, because the drug remains in the tubules for a longer period of time, may also boost its antibacterial efficacy [1]. According to the findings of earlier in vitro investigations, NCH exhibited better antibacterial activity than traditional CH in a culture medium [3]. Because of their small dimensions, nanoparticles can efficiently penetrate the intricate anatomy of the root canal system.

NAg is biocompatible and broad-spectrum, releasing silver ions. Despite being an inactive substance in bulk, it gets ionized by moisture, changing it into an extremely reactive state. By breaking down the bacterial cell envelope, blocking metabolic enzymes, and producing active oxygen species, NAg has bactericidal effects. Additionally, it collects in the pits on the cell surface and causes denaturation of the cell membrane, which results in cell death and lysis [11].

NAg also exhibits a deeper penetration than traditional CH. The possible application of NCH in combination with NAg as a root canal medication was emphasized by Afkhami et al. [14]. Material penetration into tubules is influenced by a variety of parameters, including the removal of the smear layer, the size of the radicular canal, and the chemical and physical characteristics of the material. These characteristics may have contributed to the maximum tube penetration that was attained by combining NAg and NCH [11].

Compared to other nanogroups, NCS demonstrated a lower penetration depth; this could be because of its gelling-out property, which prevents it from penetrating dentinal tubules. Compared to NCS with NAg, NCS demonstrated a greater penetration depth. Despite having smaller particles, NCS and NCS with NAg showed limited penetration depth, which may be related to NCS's propensity to aggregate. In a prior investigation, the gelling-out character and uneven dispersion of nanoparticles were noted in NCS [15].

The findings of the current study demonstrated that using conventional CH resulted in decreased fracture resistance, followed by NCH in conjunction with NAg, which is statistically significant. The most plausible explanation is that the proteoglycans and acid proteins that serve as a bonding medium between the collagen matrix and the crystals of hydroxyapatite in dentine were neutralized, dissolved, or denatured, which is caused by the significant alkalinity of CH [16]. Previous research has documented a reduction in the resistance of teeth to fracture when treated with CH [17,18], which is consistent with our findings. Our current study's findings are consistent with research by Naseri et al., who observed that NCH decreased the root dentin microhardness less than CH did [19]. There was a statistically significant difference between the two drugs.

Since NCS possesses mucoadhesive qualities, it is necessary to find out if it has any adhesive qualities comparable to those of root canal dentin. This characteristic might help to prolong the effect of intracanal medicament within the root canal [20]. Additional in vitro research is required to confirm these findings.

The coronal sections showed the greatest mean penetration depth, followed by the middle section and the lowest in the apical third (p < 0.05), which is consistent with a previous study [3]. This may be explained by the anatomical complexity of the apical third of the root canal, which includes occluded tubules, sclerotic dentin, and a lower density of tubules than in the coronal dentin. [9]. The decreased penetrability of intracanal medicaments in the apical third may have been caused by numerous anatomical factors, including blocked tubules, sclerotic dentin, and reduced tubule density. The efficiency of removal of the smear layer in the apical third of the root also diminishes, thereby reducing the penetration of medicaments.

Limitations

One of the limitations of this study is that experimental in vitro investigations don't accurately represent the state of teeth with a functional periodontium in vivo. Additional in vivo research is required to validate the results of this study.

## Conclusions

The combination of NCH and NAg had a deeper depth of penetration than any other group. Among all groups, the coronal part of the root dentin showed the greatest depth of intracanal medication penetration, followed by the middle section, and minimum penetration in the apical portion of root dentin. The fracture resistance was higher in the NCS group compared to all the other groups.
